# Toxicities of Immunosuppressive Treatment of Autoimmune Neurologic Diseases

**DOI:** 10.2174/157015911796557939

**Published:** 2011-09

**Authors:** Enrico C Lallana, Camilo E Fadul

**Affiliations:** Neuro-Oncology Program, Norris Cotton Cancer Center Dartmouth-Hitchcock Medical Center and Departments of Neurology and Medicine, Dartmouth Medical School, One Medical Center Drive Lebanon NH 03756, USA

**Keywords:** Immunosuppressive therapy, toxicity, auto-immune neurologic disease.

## Abstract

In parallel to our better understanding of the role of the immune system in neurologic diseases, there has been an increased availability in therapeutic options for autoimmune neurologic diseases such as multiple sclerosis, myasthenia gravis, polyneuropathies, central nervous system vasculitides and neurosarcoidosis. In many cases, the purported benefits of this class of therapy are anecdotal and not the result of good controlled clinical trials. Nonetheless, their potential efficacy is better known than their adverse event profile. A rationale therapeutic decision by the clinician will depend on a comprehensive understanding of the ratio between efficacy and toxicity. In this review, we outline the most commonly used immune suppressive medications in neurologic disease: cytotoxic chemotherapy, nucleoside analogues, calcineurin inhibitors, monoclonal antibodies and miscellaneous immune suppressants. A discussion of their mechanisms of action and related toxicity is highlighted, with the goal that the reader will be able to recognize the most commonly associated toxicities and identify strategies to prevent and manage problems that are expected to arise with their use.

## INTRODUCTION

Advances in our understanding of the intricate interaction between the immune system and the nervous system have translated into the routine use in clinical practice of immune suppressive therapy for neurologic disease of presumed or confirmed autoimmune etiology. Diligent research has led to therapies that can alter or modify the course of the disease in multiple sclerosis (MS), myasthenia gravis (MG), acute and chronic demyelinating polyneuropathies, and other neurologic illnesses (Table **1**). The neurologist must be aware not only of the potential benefit but also of the side effect profile to utilize the appropriate immune suppressive drug with the optimal therapeutic ratio for the individual patient.

In this review, we will discuss the medication-related toxicity of immunosuppressive treatments most widely used in neurology, as well as new medications that are advanced in the drug development pipeline with potential for Food and Drug Administration (FDA) approval. Due to the large number of therapies and side effects, an exhaustive discussion of all medications is beyond the scope of this article. We will classify the drugs according to their professed mechanism of action, briefly review their indication in neurologic disease, and then describe the most frequent adverse effects including, when appropriate, recommendations for monitoring and management (Table **2**).

## CORTICOSTEROIDS

Corticosteroids depress the immune system by stabilizing lysosomal membranes, decreasing migration of leukocytes, reducing the number of immune cells, and inhibiting the production of cytokines and other immune modulators [1]. Being the most widely used immunomodulatory medication in neurology, they are prescribed for MS, MG, chronic inflammatory demyelinating polyradiculoneuropathy (CIDP), central nervous system (CNS) vasculitides and neurosarcoidosis to name a few.

Corticosteroid side effects depend on daily dose, dosing frequency, and duration of treatment. Brief courses of these drugs, even at very high doses are typically well tolerated. Prolonged use can cause a multitude of symptoms that range from skin changes to fatal adrenal suppression. The most common side effects of chronic use include: hypertension, cushingoid habitus; skin changes; proximal myopathy; psychological disturbances; hyperglycemia and diabetes; peripheral edema; acid peptic disease; infections; osteoporosis and aseptic necrosis of bone. Patients on chronic treatment should be monitored for osteoporosis and will frequently require of the use of bisphosphonates. Dosing corticosteroids treatment on alternate days is thought by many to be associated with fewer side effects but there have been no good comparative trials to prove this claim.

## CYCLOPHOSPHAMIDE

As an alkylating agent, cyclophosphamide interferes with the growth of rapidly proliferating cells, including lymphocytes, by intercalating DNA [2]. It is used off-label in refractory MS, neurosarcoidosis and CNS vasculitides.

Cyclophosphamide therapy is associated with nausea and vomiting, hair loss, skin irritation, liver enzyme abnormalities and marrow depression. Infertility occurs in both men and women and the reported incidence of amenorrhea is as high as 42% with 24% becoming permanent [3].

A potentially life threatening side effect is the development of acute hemorrhagic cystitis. Acrolein, a metabolite of cyclophosphamide, enters the uroepithelial cells and activates intracellular pathways which result in peroxynitrite production that ultimately leads to necrotic cell death [4, 5]. The use of intravenous fluid hydration and prophylactic mesna, a chelating agent for acrolein, has significantly lowered the incidence of this complication [6, 7]. However, since cyclophosphamide doses used in MS are less than 1g/m^2^, there is usually no need for prophylactic mesna in this setting.

Bladder cancer, also thought to be due to accumulation of acrolein, has an estimated incidence of 5% at 10 years and 16% at 15 years [8]. A twenty year follow-up study showed that those who developed cancer in relation to cyclophosphamide had received a higher dose of the medication and that it can occur many years after its discontinuation [9]. Cardiac side effects have been observed after high doses of cyclophosphamide (exceeding 200 mg/kg) and the abnormalities range from mild electrocardiographic changes to fatal cardiomyopathy [10, 11]. Congestive heart failure and pericarditis may occur within the first 10 days of treatment. The risk factors include prior anthracycline/anthracenedione therapy, age >50 years or chest irradiation [10, 12]. While these risk factors are not absolute contraindications, caution must be used when considering cyclophosphamide therapy in these patients.

## MITOXANTRONE

Mitoxantrone is an anthracenedione that crosslinks DNA, interferes with RNA synthesis and inhibits the enzyme topoisomerase II [13]. It is approved by the FDA for reducing neurologic disability and/or the frequency of clinical relapses in patients with secondary (chronic) progressive, progressive relapsing, or worsening relapsing-remitting MS.

The most common serious toxicity is myelosuppression that appears about 10 -14 days after treatment. Leucopenia is more frequently seen than thrombocytopenia and anemia. Transaminase elevation is noted in up to 15% of patients but this is typically transient. Secondary amenorrhea occurs in up to 25% of MS patients [14]. Mitoxantrone is moderately emetogenic, and rarely causes mucositis and hair loss.

A serious side effect of mitoxantrone is cardiotoxicity that usually appears after large cumulative doses. Drug-related congestive heart failure occurs in 2.6–6.0% of patients who received cumulative doses of up to 140 mg/m^2^ as treatment for leukemia or solid tumours [15]. The incidence of asymptomatic left ventricular ejection fraction (LVEF) that is <50% is approximately 5% in MS. When treatment is limited to a cumulative lifetime dose of 60 mg/m^2^, the observed incidence is <0.2% [16]. Due to these complications, mitoxantrone must not be given to patients with underlying cardiac disease especially cardiomyopathy, with LVEF <50%, who previously received treatment with other anthracyclines or had mediastinal irradiation. The FDA recommends quantitative evaluation of LVEF prior to each treatment and yearly after its completion. A recent study suggests that measurement of brain natriuretic peptide in blood may be a useful marker of sub-clinical cardiac injury [17].

Topoisomerase II inhibitors such as mitoxantrone have also been associated with the development of secondary leukemia, most frequently acute myeloid leukemia [18]. The early experience in MS suggested a low incidence of the complication, estimated at 0.21%[19-20] but recent studies suggest a higher incidence of up to 3.3% [21]. Most cases have occurred in patients receiving >60 mg/m^2^ of mitoxantrone. There was also an initial assumption that the prognosis of treatment-associated leukemia was better [22], but some authors report a mortality rate as high as 24% [23]. A post marketing report found 39 cases of secondary leukemia in the United States from 2003 to 2007. Most of the cases were either acute myeloid leukemia (38.5%) or acute promyelocytic leukemia (33.3%). While these reports were of a spontaneous nature and the incidence rate cannot be estimated, there needs to be an increased vigilance towards the occurrence of secondary leukemia.

## INTRAVENOUS IMMUNOGLOBULIN

Intravenous immunoglobulin (IVIg) is prepared from pooled plasma and has a complex mode of action that is not completely defined. It is thought to modulate the expression and function of Fc receptors, interfere with the activation of complement and the cytokine network, produce antiidiotypic antibodies, and affect the functions of lymphocytes [24]. IVIg is indicated in the treatment of CIDP, and is used off-label in Guillain-Barre syndrome, MG, multifocal motor neuropathy, and dermatomyositis.

About 10% of those receiving IVIg experience side effects that are generally mild [25]. The most common are headaches, chills, myalgias, and chest discomfort that occur during infusion. Less frequent side effects are dyspnea, back pain, nausea, vomiting, diarrhea, blood pressure changes, and tachycardia that are usually transient and subside without intervention. After the infusion, patients sometimes report nausea, loss of appetite, fatigue, and fever. Skin reactions of uncertain etiology are also known to occur within 24 hours after infusion [26]. Pre-treatment with analgesics, non-steroidal anti-inflammatories, antihistamines, or low-dose corticosteroids has been frequently observed to be beneficial in preventing these reactions.

Aseptic meningitis is an infrequent adverse reaction of immunoglobulin therapy and has been reported to have an incidence as high as 11% [27] although most reports estimate it to be 1% [26]. It occurs within 24 hours of infusion and can last 3-5 days before spontaneously resolving.

There is a risk for a severe anaphylactic reaction in patients who have IgA deficiency. The anti-IgA antibodies in the serum of patients with this deficiency form an immune complex with the traces of IgA in the infused IVIg. Checking for IgA levels can screen patients at risk and the use of IgA depleted IVIg can prevent this reaction [28].

Elderly patients, poorly hydrated individuals and patients with diabetes or impaired kidney function are at risk for acute renal failure [26]. Additionally, patients who receive high doses or high infusion rates of IVIg or have a history of prior thromboembolism are at risk of thromboembolic events [29].

The possibility of transmission of infectious agents is present whenever IVIg is used although the occurrence is rare. The use of IVIg remains relatively safe compared with other immunosuppressive drugs or interventions.

## AZATHIOPRINE

Azathioprine, a prodrug of 6-mercaptopurine (6-MP), interferes in purine nucleotide synthesis and metabolism which makes it an effective inhibitor of lymphocyte proliferation. It has been used off-label in MS and MG.

This medication causes nausea and vomiting in about 22% of patients with a third having severe enough symptoms to cause its discontinuation [30]. These side effects start soon after initiation of treatment. Pancreatitis and hepatotoxicity have also been reported and these symptoms occur within the first 3-6 months [31, 32]. There are frequent liver enzyme elevations that resolve spontaneously with dose decreases or discontinuation.

Monitoring of complete blood counts is recommended during azathioprine therapy due to leucopenia and thrombocytopenia. In the rheumatology literature, the incidence of leucopenia is about 27% and thrombocytopenia 5% [33, 34]. Dosage adjustments are necessary when leucocyte count becomes lower than 3000/uL. Macrocytic anemia has been reported and is treated with folate supplementation [35]. Thiopurine S-methyltransferase (TPMP) catalyzes 6-MP to its inactive metabolite methyl-6-mercaptopurine. Patients who are deficient of TPMP may be more sensitive to the myelosuppressive effects. Additionally, patients on concurrent therapy with drugs which may inhibit TPMP (olsalazine) or xanthine oxidase (allopurinol) may be susceptible to the same sensitivity. A high incidence of azathioprine intolerance in MS patients has been reported which is purportedly secondary to genetic polymorphysism in the enzymes metabolizing the drug [36]. 

**Fig. (1):**
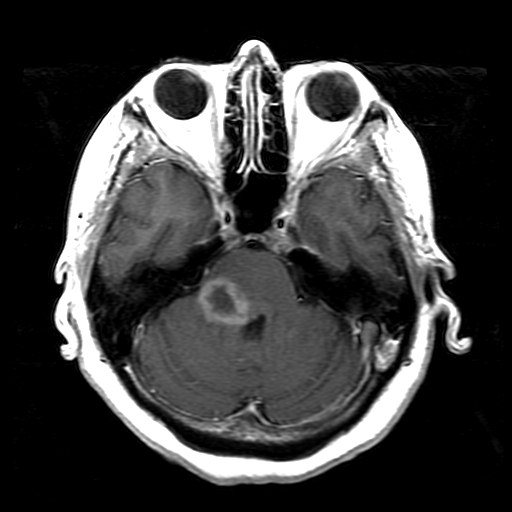
A 54 year old woman with auto-immune hepatitis on treatment with azathioprine for five years who presented with sub-acute neurologic symptoms of right facial drooping and loss of vision in the right eye. MRI revealed a ring-enhancing pontine lesion. Pathological examination proved this to be large B-cell primary central nervous system lymphoma (PCNSL). The imaging characteristics of the tumor were that of a ring-enhancing mass typical of PCNSL found in HIV patients.

Malignancies are known to occur in azathioprine treated patients but the exact incidence is unknown. Although originally thought to be due to the immune suppression caused by the drug, several studies have shown that azathioprine has a direct mutagenic effect [37-39]. Lymphoma (Fig. **1**) is the most well known of these rare malignancies and is estimated to occur in 0.5% of renal transplant patients [40]. Other tumors associated with azathioprine are: squamous cell carcinomas of the skin, Kaposi's sarcoma, in situ carcinomas of the cervix, carcinomas of the vulva and perineum, hepatobiliary carcinoma, and mesenchymal tumors [41, 42]. 

Serious infections are a constant hazard for patients receiving azathioprine. The overall infection rate is estimated to be approximately 9% [34]. Fungal, viral, bacterial, and protozoal infections have been observed and must be treated with the appropriate antimicrobial therapy.

## MYCOPHENOLATE

This drug inhibits purine synthesis and has mostly been used off-label in MG [43, 45, 46]. Mycophenolic acid inhibits the enzyme inosine monophosphate dehydrogenase, and as a consequence, it decreases de novo guanosine nucleotide synthesis. Since lymphocytes are dependent on the de novo synthesis of purines, this deficiency results in significant reduction of lymphocyte proliferation. Mycophenolate has also been shown to directly induce programmed cell death of activated human T cells and inhibit the antibody production of activated B cells [47, 48]. As a result, lymphopenia is the most common adverse reaction. At therapeutic doses, lymphopenia is noted in about 2% of myasthenics [49]. Anemia, neutropenia and thrombocytopenia are also seen [50]. These hematologic side effects are easily reversed with lowering the dosage or discontinuing the drug. 

Mycophenolate causes nausea, vomiting, diarrhea and cramping but these side effects are self-limited and do not usually cause discontinuation of treatment [51]. In a prospective study of patients with MG, the incidence of mild nausea was 8% and severe gastrointestinal symptoms 1% [49]. Gastrointestinal bleeding is occasionally seen in transplant patients receiving mycophenolate but has not been reported in rheumatologic or neurologic literature. This medication has been associated with transient elevations of liver enzymes but no other significant hepatotoxicity [52].

There have been case reports of lymphoma and other malignancies associated with mycophenolate treatment. However, in a prospective observational study of renal transplant patients treated with mycophenolate, there was no evidence of increased risk for malignancy relative to other immunosuppressive treatments [53]. This suggests that the increased rate of malignancy is associated with the immune suppression and not a direct mutagenic effect.

Mycophenolate treatment can increase the risk of opportunistic infections such as activation of latent viral infections. Progressive multifocal leukoencephalopathy (PML) has been reported and presents with hemiparesis, apathy, confusion, cognitive deficiencies, visual changes and ataxia [54].

## CLADRIBINE

Cladribine is another medication that inhibits purine synthesis. It is an adenosine deaminase-resistant purine nucleoside [55] that causes lymphocyte apoptosis [56]. A recent trial of oral therapy in patients with relapsing–remitting MS revealed a significantly lower annualized rate of relapse, higher relapse-free rate, lower risk of 3-month sustained progression of disability and significant reductions in the brain lesion count on magnetic resonance imaging (MRI) [57].

The most common and serious side effect of the oral administration is lymphopenia, although moderate to severe neutropenia, thrombocytopenia and pancytopenia may also occur.

Reactivation of herpes zoster, varicella and tuberculosis due to the immune suppression have been reported. Mild respiratory tract infection, urinary tract infection, and subcutaneous abscess have also been observed. Hepatobiliary disorders like cholelithiasis, infectious hepatitis and cholecystitis were also seen. There were three cases of cancer: a melanoma and carcinomas of the pancreas and ovary. Peripheral neuropathy, a known rare side effect of the injectable cladribine, has not been reported with the oral preparation.

## CYCLOSPORINE

Cyclosporine, a polypeptide immunosuppressant agent [58], preferentially inhibits clonal expansion of activated T-helper cells while allowing the activation and expression of T-suppressor lymphocytes [44]. The drug binds to the cytosolic protein cyclophilin to form a complex that inhibits calcineurin, an inducer of the production of interleukin-2. It is used off-label in the treatment of MG and MS.

The most common limiting adverse event of this medication is nephrotoxicity, both acute and chronic, which was first recognized in renal transplant patients. The acute nephrotoxicity is usually reversible and believed to be due to a vasoconstriction of the afferent arterioles [59]. The irreversible chronic form is a consequence of interstitial fibrosis that develops after 6 to 12 months of treatment [60] and increases the risk of end-stage renal disease and need for hemodialysis. Since nephrotoxicity is dose dependent and cyclosporine has a narrow therapeutic range, blood concentration should be regularly monitored. However, drug monitoring does not always prevent nephrotoxicity. Renal function monitoring by laboratory parameters is required and at the earliest signs of nephrotoxicity, the medication should be discontinued.

Hypertension is a common side effect that is easily managed with medication. Potassium-sparing diuretic agents are avoided due to the occasional hyperkalemia associated with cyclosporine use. Additionally, calcium channel blockers, which may have some beneficial effect on renal afferent arteriolar blood flow, can interfere with cyclosporine metabolism and its use is not recommended in this group of patients.

Infrequent mild to moderate adverse events include hirsutism, tremor, gum hyperplasia, diarrhea, cramps, anorexia, confusion and paresthesias. Similar to other immunesuppressants, there is a recognized increase in susceptibility to infection and possible development of lymphoma and other neoplasms [61].

## TACROLIMUS

This is another immune suppressive medication that inhibits, by an unknown mechanism, T-lymphocyte activation. Like cyclosporine, it has a macrolide molecule and inhibits calcineurin. Its main use has been in organ transplantation and while its main limiting factor is also nephrotoxicity, it has not been a significant issue in the published studies treating patients with MG [62-67]. In these studies, there were only sporadic cases of treatment limiting side effects. There was one case of elevated serum creatinine in the setting of hypertension [65], one case of severe headache, and one case of severe eye pain [63]. However, there were three cases of malignancy which may or may not be related to the treatment since these occurred within 4 to 6 months of initiating therapy and patients in this study had previously been chronically treated with cyclosporine [67].

Other side effects include hypomagnesemia, paresthesias, tremor, diarrhea, constipation, hypertension, abdominal pain, and hyperkalemia. Severe infections have been reported in transplant patients but were not observed to be significant in MG studies.

Tacrolimus appears to be a well tolerated treatment but close observation of patients on this medication is necessary because of its potential for serious side effects.

## NATALIZUMAB

Natalizumab is a humanized monoclonal antibody that targets the alpha-4 subunit of integrins on the surface of lymphocytes. This interaction prevents the integrin from binding to the endothelial receptor, vascular-cell adhesion molecule-1 [68], thereby effectively blocking the transmigration of lymphocytes across the blood-brain barrier. When used in patients with relapsing MS, it reduces the risk of the sustained progression of disability and the rate of clinical relapses [69, 70].

The most serious adverse event associated with its use is PML. The first 2 reports of PML were seen in patients who had received natalizumab in combination with intramuscular interferon beta 1a [71, 73]. Following these two events, a review of all patients treated revealed that there was a third case of PML in a Crohn's disease patient who had been heavily pretreated with immunosuppressants [72]. This prompted the manufacturer to voluntarily withdraw the medication from the market in February, 2005. After review of safety information and no further occurrences of PML, a prescription safety risk monitoring system was devised and the medication was allowed to return to the market in June, 2006. At that time, PML was believed to occur only in immunosuppressed individuals or if used in combination therapy with an immunomodulator. In June, 2008, one of the first cases of PML in MS patients treated with natalizumab monotherapy was found. The FDA reports that as of January 21, 2010 there have been 31 confirmed cases of PML. Despite the number of cases, the FDA believes that its clinical benefits continue to outweigh the potential risks. Based on their information, there have been no reports of PML in patients treated for less than 12 months and that the incidence of PML in patients with at least 24 months of treatment is about 1 per 1000 patients [74].

JC virus infection of oligodendrocytes in an immune suppressed state is the cause of PML. The virus is present in 58% of the general population, indicating asymptomatic current or previous infection [75]. In MS patients, it is believed that natalizumab reduces immune surveillance in the CNS, thereby causing regional immune compromise. The virus is reactivated from the kidney or lymphatic tissue which is then disseminated into the CNS. However, other authors believe that the JC virus is ubiquitous and is also latent in the brain [76]. Prior to natalizumab, PML was virtually unseen in the MS population.

**Fig. (2):**
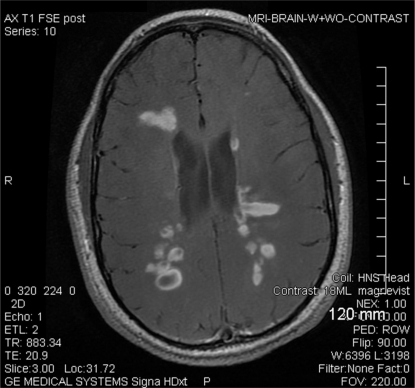
A 25 year old man with a diagnosis of MS had sudden change in neurologic status after discontinuation of natalizumab. He had been on therapy for two years with good response but natalizumab was discontinued due to concerns of increased risk for PML. He switched to glatiramer acetate but after three months from discontinuation of natalizumab, he had acute symptoms of ataxia and fatigue. MRI revealed multiple new T2/FLAIR hyperintensities and T1 enhancing lesions. He was treated with a 5 day course of methylprednisolone and his symptoms promptly resolved (case report and MRI courtesy of Ann Cabot, D.O.).

Clinically, the symptoms are difficult to distinguish from a severe MS relapse. Once PML is suspected, natalizumab must be discontinued and MRI should be done immediately. However, there is not one specific PML MRI appearance. Most commonly, PML appears as a large white matter T2/FLAIR hyperintensity that can involve the adjacent gray matter without producing any significant mass effect. Polymerase chain reaction (PCR) analysis of cerebrospinal fluid for JC virus DNA is very sensitive and specific for the diagnosis [77, 78]. In cases where suspicion remains strong despite a negative cerebrospinal fluid PCR, a brain biopsy may be indicated. Once the diagnosis is confirmed, immune reconstitution must be facilitated. Various antiretroviral drugs [79, 80] have been used, as well as other medications such as mefloquine and mirtazapine [81] but evidence of their efficacy is lacking. Several authors have advocated plasma exchange and immunoabsorption to remove any remaining natalizumab in the patient's circulation [82]. However, abrupt immune reconstitution may precipitate a severe immune reconstitution inflammatory syndrome (IRIS) which is manifested by worsening symptoms and MRI abnormalities [83]. An IRIS-like rebound phenomenon can occur without intervention, as exemplified by the case described (Fig. **2**). Other diseases associated with CNS immune suppression like toxoplasmosis [84], primary central nervous system lymphoma [85] and melanoma [86] have been reported but a clear association has not been established.

Hepatic injury has been reported in several patients and in one case series [87], half of the cases had features of autoimmune hepatitis. So far, there have been no atypical infections that have been documented to affect the liver during natalizumab therapy.

The more common side effects of natalizumab are fatigue and allergic reactions. Headaches, chest discomfort, rigors and syncope have also been frequently reported. The symptoms are typically noted during the infusion or immediately after. Mild to moderate infections have been observed but there were no significant differences between the natalizumab and the placebo treatment groups in the original pivotal trials [69, 70].

## RITUXIMAB

Rituximab is a monoclonal antibody that binds to the CD20 antigen in mature B lymphocytes. It causes depletion of its target cells through a combination of cell-mediated and complement-dependent cytotoxic effects and the promotion of apoptosis [88, 89]. Several preliminary studies have shown that rituximab can reduce inflammatory brain lesions and clinical relapses in MS [90, 91]. There are several case reports of its efficacy in refractory MG [92-97], CIDP [98-101] and CNS involvement of systemic vasculitis [102, 103]. Ocrelizumab is a humanized monoclonal antibody similar to rituximab that is currently in phase II trials for the treatment of relapsing MS.

These antibodies have also been associated with PML. Although the original cases were in patients with non-Hodgkin's lymphoma who were receiving chemotherapy, two cases of PML occured in patients with systemic lupus erythematosus [104]. A third case was reported to the FDA in 2009 in a patient with rheumatoid arthritis [105]. It is estimated that the rate of occurrence is about 2.2 per 100,000, significantly lower than what is seen in natalizumab.

Serious, sometimes fatal infections have been reported. Hepatitis B reactivation resulting in hepatic failure, fulminant hepatitis and death have been seen in rituximab treated patients with hematologic malignancies [106]. In a phase II trial for MS, there were also mild to moderate infections of the upper respiratory tract, urinary tract and sinuses [90]. Frequently reported mild side effects include chills, headache, nausea, pruritus, fever, fatigue, and throat irritation.

## ALEMTUZUMAB

Alemtuzumab is a humanized monoclonal antibody that targets the CD52 receptor, causing mononuclear cell depletion that can last for years [107]. In a prospective phase II MS study, it significantly reduced the rate of sustained accumulation of disability and annualized rate of relapse when compared against interferon beta 1a [108].

Paradoxically, autoimmunity is one of the most serious side effects of alemtuzumab, probably associated to regulatory lymphocyte depletion. Immune thrombocytopenic purpura occured in 6 patients resulting in one death during the above referenced phase II trial [108]. Autoimmune thyroid disease was frequently found in the alemtuzumab treatment group and was associated with thyroid autoantibodies in 96% of affected individuals. Graves' disease is associated with increased numbers of CD8^+^ cells and a low production of memory CD4^+^ cells 15-18 months after treatment [109]. These findings are consistent with previously established association of CD8^+^ positive counts in Graves' disease [110].

It frequently causes severe and persistent lymphopenia along with moderate to severe neutropenia, anemia and thrombocytopenia. Anaphylactoid infusion reactions which include pyrexia, chills, hypotension, urticaria, and dyspnea, were common but are a rare cause of discontinuation of treatment. Abnormal liver function tests are occasionally seen and are usually reversible.

Mild to moderate infections of the upper respiratory tract were common in the MS trial. There have been no reported cases of PML to date.

## FINGOLIMOD

The immune modulating drug FTY720 (fingolimod) induces lymphopenia by revesibly redistributing lymphocytes from the circulation to the secondary lymphoid tissues. It is thought to act through sphingosine 1-phosphate (S1P) signaling pathways to modulate chemotactic responses and regulate the recirculation of lymphocytes [111, 112]. In two phase III studies, it was shown to decrease the relapse rate, disability progression and number of new or enlarged lesions on T2 -weighted images, gadolinium-enhancing lesions, and brain-volume loss in patients with relapsing MS [113, 114]. 

The S1P receptor also regulates heart rate [115], coronary artery blood flow [116] and blood pressure [117]. Atrial myocytes have the S1P3 receptors that fingolimod effectively signals [112]. This interaction results in slowing of the sinoatrial node and reactivation of G-protein-activated potassium channels 1 and 4. It is also theorized that fingolimod has similar effects on the atrioventricular (AV) node. Predictably, the most common adverse event in the clinical trials were bradycardia and AV block. The transient, dose dependent decrease in heart rate occurred within one hour of the first dose of the medication. The majority of the patients who had bradycardia were reported to be asymptomatic while a few had symptoms that resolved within 24 hours without intervention [114, 118].

The most frequently reported infections involved the upper respiratory and urinary tracts. Severe infections were seen, including herpes virus reactivations. There is also one case report of hemorrhagic focal encephalitis complicated by complex partial seizures after 7 months of treatment [119].

The most common side effects are mild and include headache, fatigue, dizziness, musculoskeletal pain, cough and dyspnea. There are few reports of elevation of liver function tests, hypertension and leucopenia.

## CONCLUSION

The treatment of presumed autoimmune neurologic diseases has rapidly expanded in the last two decades. The research to date has resulted in the acceptance of immune suppressive drugs as an important regimen for neurologic diseases. Prior to the advent of these medications, corticosteroids were used widely, in some cases with little proven benefit, resulting in a high rate of long term side effects. Since then, the use of IVIg, mycophenolate, azathioprine, cyclosporine and tacrolimus has provided a better therapeutic ratio than corticosteroids in neuromuscular disease and neurosarcoidosis. Cytotoxic chemotherapy has offered patients with MS, CNS vasculitis and neurosarcoidosis a therapeutic option when they become refractory to first line agents. Antibodies and other targeted biologic agents have the potential for improved disease control in MS, as well as in other autoimmune neurologic illnesses. However, it must be pointed out that only mitoxantrone, natalizumab and IVIg have FDA approved indications in neurologic diseases. Great care and diligence must be made whenever deciding to use these medications as recent clinical trials have taught us about unexpected adverse events. As we have outlined here, there can be significant problems associated with these treatments, the most important of which are hematotoxicity, increased infection rate, and carcinogenicity. With this knowledge we proceed with vigilance but we anticipate that the advances in our understanding of the immune system will bring more targeted therapies that will not only result in improved efficacy but also decreased toxicity. 

## Figures and Tables

**Table 1:** Immunosuppressive Medications Used for Treatment of Autoimmune Neurologic Diseases

Immunosuppressive Therapy	FDA Approved Indication	Generally Accepted Indication	Investigational
Cyclophosphamide	*Leukemia, Lymphoma, carcinoma*	Neurosarcoidosis, MS, Primary or secondary CNS vasculitis	
Mitoxantrone	MS, *Prostate cancer*		
Intravenous immunoglobulin	CIDP	MG, LEMS, GBS, MMN	
Azathioprine	*Renal transplantation, Rheumatoid arthritis*	MG	CIDP, Neurosarcoidosis
Mycophenolate	*Renal, hepatic and cardiac transplantation*	MG	CIDP, Neurosarcoidosis
Cyclosporine	*Renal, hepatic and cardiac transplantation*	MG	MS
Tacrolimus	*Renal, hepatic and cardiac transplantation*	MG	
Cladribine	*Hairy cell leukemia*		MS, Neurosarcoidosis
Natalizumab	MS, *Crohn’s disease*		
Rituximab	*Non-Hodgkin’s lymphoma, Rheumatoid arthritis*		MS, CIDP, CNS vasculitis
Alemtuzumab	*B-cell chronic lymphocytic leukemia*		MS
Fingolimod			MS

MS- Multiple sclerosis, MG – Myasthenia gravis, GBS – Guillain-Barre Syndrome, CIDP - Chronic inflammatory dyemelinating polyradiculoneuropathy, LEMS – Lambert-Eaton myasthenic syndrome, MMN – Multifocal motor neuropathy CNS – Central nervous system

**Table 2:** Toxicities of Immunosuppressive Medications

Drug	MOA	Major Toxicity
Cytotoxic chemotherapy		
Cyclophosphamide	Intercalates DNA	Leucopenia, hemorrhagic cystitis, bladder malignancy, myeloproliferative disorders, Infertility
Mitoxantrone	Intercalates DNA, interferes with RNA synthesis	Cardiomyopathy, leukemia (myeloproliferative), Infertility
Chemotherapy Immunosuppresant		
Azathioprine	Inhibition of purine nucleotide synthesis	Leucopenia, pancreatitis, hepatotoxicity, malignancy
Mycophenolate	Inhibition of guanosine nucleotide synthesis	Gastrointestinal symptoms, lymphopenia, infections
Cladribine	Inhibition of purine nucleotide synthesis	Lymphopenia
Monoclonal antibody – transmigration inhibition		
Natalizumab	Prevents trans-migration of lymphocytes across the blood-brain barrier	PML and other opportunistic infections
Monoclonal antibody – Lymphocyte depletion		
Rituximab	Depletion of B-lymphocytes	PML and other opportunistic infections
Alemtuzumab	Depletion of lymphocytes	Autoimmune disease (ITP, Grave’s disease), leucopenia
Lymphocytic sequestration		
Fingolimod	Lymphatic sequestration of lymphocytes	Bradycardia, infection
Non-specific antibody binder		
Intravenous immunoglobulin	Modulation of the expression and function of Fc receptors	Aseptic meningitis, anaphylaxis, renal failure

MOA - Mechanism of action, PML - Progressive multifocal leukoencephalopathy, ITP - Immune thrombocytopenic purpura.
